# Positive allosteric modulator activity of ginsenosides is restricted to P2X7 and P2X4 receptors

**DOI:** 10.1007/s11302-026-10162-6

**Published:** 2026-05-23

**Authors:** Elizabeth Allum, Leanne Stokes

**Affiliations:** School of Chemistry, Pharmacy & Pharmacology, University of East Anglia, Norwich Research Park, Norwich, NR4 7TJ UK

**Keywords:** ATP, P2X receptor, Ginsenoside, Membrane potential

## Abstract

Positive allosteric modulators of P2X7 receptors hold promise as host-directed immune enhancers in intracellular infections. We have previously reported the use of protopanaxadiol ginsenosides as positive allosteric modulators of human, rat and mouse P2X7 receptors plus human P2X4 receptors, however, the effect of ginsenosides on other P2X receptors is unknown. Here, we screened a range of ginsenosides and related glycosides at hP2X1, hP2X2 and hP2X3 receptors expressed in HEK-293 cells utilising a membrane potential assay. Our results demonstrate the utility of the membrane potential assay across all P2X receptors and the lack of significant potentiation of ATP-induced responses at hP2X1, hP2X2 and hP2X3 receptors by ginsenosides and related glycosides. We report that S-Rg3 also acts as a positive allosteric modulator at hP2X4 receptors in addition to hP2X7 receptors. This information is important for determining the selectivity of ginsenoside positive allosteric modulators for P2X7 and P2X4.

## Introduction

The seven subtypes of P2X receptors are structurally similar with a common trimeric structure [1]. Subtle differences exist in the orthosteric ligand binding site, giving rise to different affinities for ATP and differences in pharmacological activity of ATP analogues (e.g. BzATP, αβ-meATP) [2]. Allosteric modulators can interact with several sites on P2X receptors, identified through a number of x-ray crystallography and cryo-EM studies [3–7]. One negative allosteric modulator site is located behind the orthosteric ATP site on P2X7 and an analogous site is found on P2X4 [3–6] whereas with P2X3, a negative allosteric modulator site is located beneath the orthosteric site [7]. Several negative allosteric modulators for P2X receptors have entered clinical trials for a range of disorders [2], with gefapixant being the first clinically used medicine targeting a P2X receptor [8].

The discovery and characterisation of positive allosteric modulators (PAMs) of P2X receptors lags behind that for negative allosteric modulators. Over the last decade several positive modulators have been documented, reviewed in [9] with a bias towards those acting at P2X7. Our group discovered a series of positive allosteric modulator compounds derived from *Panax ginseng* [10] and subsequently we have characterised their functional effects in macrophages [11, 12] and explored a potential binding site in the central vestibule region [13]. We found that several ginsenoside compounds could also potentiate P2X4 responses [14], suggesting that this could be a conserved mechanism of action within the family. We have also previously reported a series of related glycosides with PAM activity at P2X7 [15]. This study aims to investigate whether selected protopanaxadiol ginsenosides (most commonly found within *P. ginseng* plant extracts) could potentiate ATP responses at other P2X receptors, focusing on P2X1—3. To do this we wanted to use the same experimental assay system to minimise variation between assays therefore we employed the membrane potential assay used in several other studies [16–19].

## Methods

### Materials

Ginsenosides were purchased from ChemFaces as purified compounds, CK was purchased from CarboSynth. 10 mM stocks were prepared in DMSO and kept frozen at −20 ˚C in glass vials. ATP was purchased from Sigma Adrich (A7699) and prepared as a 100 mM stock in water with pH adjusted to 7.4 with 5 M NaOH. Aliquots were kept frozen at −20 ˚C and used only once.

### Cell culture

HEK-293 cells were used to generate stable cell lines expressing human P2X receptors. Original transfections were performed using Lipofectamine 2000 (Fisher Scientific) and cells were subjected to geneticin (800 µg/ml) selection for 2—3 weeks followed by single cell cloning by limiting dilution in 96-well plates. Individual clones were selected for further study based on ATP-induced responses. Cell lines were maintained in DMEM:F12 media supplemented with 10% foetal bovine serum (PanBiotech), penicillin (100 U/ml) and streptomycin (100 µg/ml) (Fisher Scientific) and kept in a humidified incubator with 5% CO_2_. Stable P2X-expressing cells were kept under geneticin (HelloBio) selection at 400 μg/ml. Passaging was performed twice weekly using 0.25% Trypsin–EDTA (Fisher Scientific).

### Membrane potential measurements

96-well plates (catalogue number 10212811, ThermoFisher Scientific) were coated with 50 µg/mL Poly-D-Lysine (P6407, Sigma-Aldrich) and left for at least one hour before being washed with sterile water and allowed to dry. Cells were plated in 100 µL of complete DMEM/F-12 the day prior to experimentation into PDL-coated 96-well plates at 2—3 × 10^4^ cells per well. Plated cells were kept in a humidified 5% CO_2_ incubator at 37 °C. The FLIPR Membrane Potential Assay Kit (Blue Bulk kit, Molecular Devices) was reconstituted according to the manufacturer’s instructions using standard extracellular buffer (145 mM NaCl, 2 mM KCl, 2 mM CaCl_2_, 1 mM MgCl_2_, 13 mM glucose, 10 mM HEPES, pH 7.3—7.4) and kept in 5 ml aliquots frozen at −20˚C. This was then diluted 1 in 2 with the extracellular buffer immediately prior to use unless otherwise stated. Cell medium was removed from the 96-well plate and replaced with 100 µL of membrane potential blue assay buffer. Cells were incubated in this buffer for 30 min at 37 °C in a humidified incubator with 5% CO_2_. Cells were then transferred to a FlexStation 3 plate reader (Molecular Devices) and warmed at 37 °C for an additional 5 min prior to experimentation. Fluorescence measurements were acquired using SoftMax Pro v5.4 software (Molecular Devices). Fluorescence emission was measured at 565 nm for 120 s with an excitation wavelength of 525 nm (3 reads per well, PMT medium, interval 2 s). Agonists and screened compounds were made up at either 5 × or 10 × final concentrations in buffer and applied to cells after 20 s of read time at an injection rate of 2—4 (31 µl/sec to 62 µl/sec). For dual injection screening experiments conducted in membrane potential blue, compounds were injected at 20 s, followed by a 60 s interval before the injection of the agonist ATP at 80 s (allowing for 60 s of pre-treatment with the compound before the application of ATP).

### Fura-2 calcium measurements

Cells were loaded with 100µL of loading buffer per well (low divalent extracellular buffer 145 mM NaCl, 2 mM KCl, 0.2 mM CaCl_2_, 13 mM glucose, 10 mM HEPES, pH 7.3—7.4) containing 250 µM sulfinpyrazone (Sigma Aldrich) and 2 µM Fura-2-acetoxymethyl (fura-2AM) calcium indicator dye (Hello Bio, UK) and incubated for 45 min at 37 °C in a humidified incubator with 5% CO_2_. This allows the membrane-permeant fura-2 AM ester to move into cells, after which it is cleaved by cellular esterases to reveal the active fura-2 Ca^2+^ indicator dye. After loading, the loading buffer was removed and replaced with 180µL per well of standard extracellular buffer. Compounds to be tested were made up at 10 × final concentration and were injected 40 s into read time at a rate of 2—4. Intracellular Ca^2+^ measurements were made using a Flexstation 3 Multimode Plate Reader (Molecular Devices) and data analysed using SoftMax Pro v5.4 software (Molecular Devices). Fluorescence emission was measured at 510 nm for 300 s (3 reads per well, PMT medium, interval 3.5 s) with dual excitation wavelengths of 340 nm and 380 nm to give a ratio between Ca^2+^-bound and Ca^2+^-free fura-2 respectively.

### Plotting and statistical analysis

Data from membrane potential experiments were plotted as area under curve (AUC) as % of control where response with each compound was normalised to the vehicle control. For fura-2 experiments, ratio data was analysed with zero baseline (3 points) and area under curve calculated from 0—300 s. Data from three independent experiments was collated and tested for statistical significance using a one-way ANOVA with Dunnett’s multiple comparison test (GraphPad Prism v6). P < 0.05 was taken as the minimum level of statistical significance. Dose–response curves were plotted by a nonlinear regression fit with variable slope using GraphPad Prism software version 6. Half-maximal responses are expressed as mean EC_50_ values with standard deviation from three independent experiments.

## Results

**Fig. 1:**
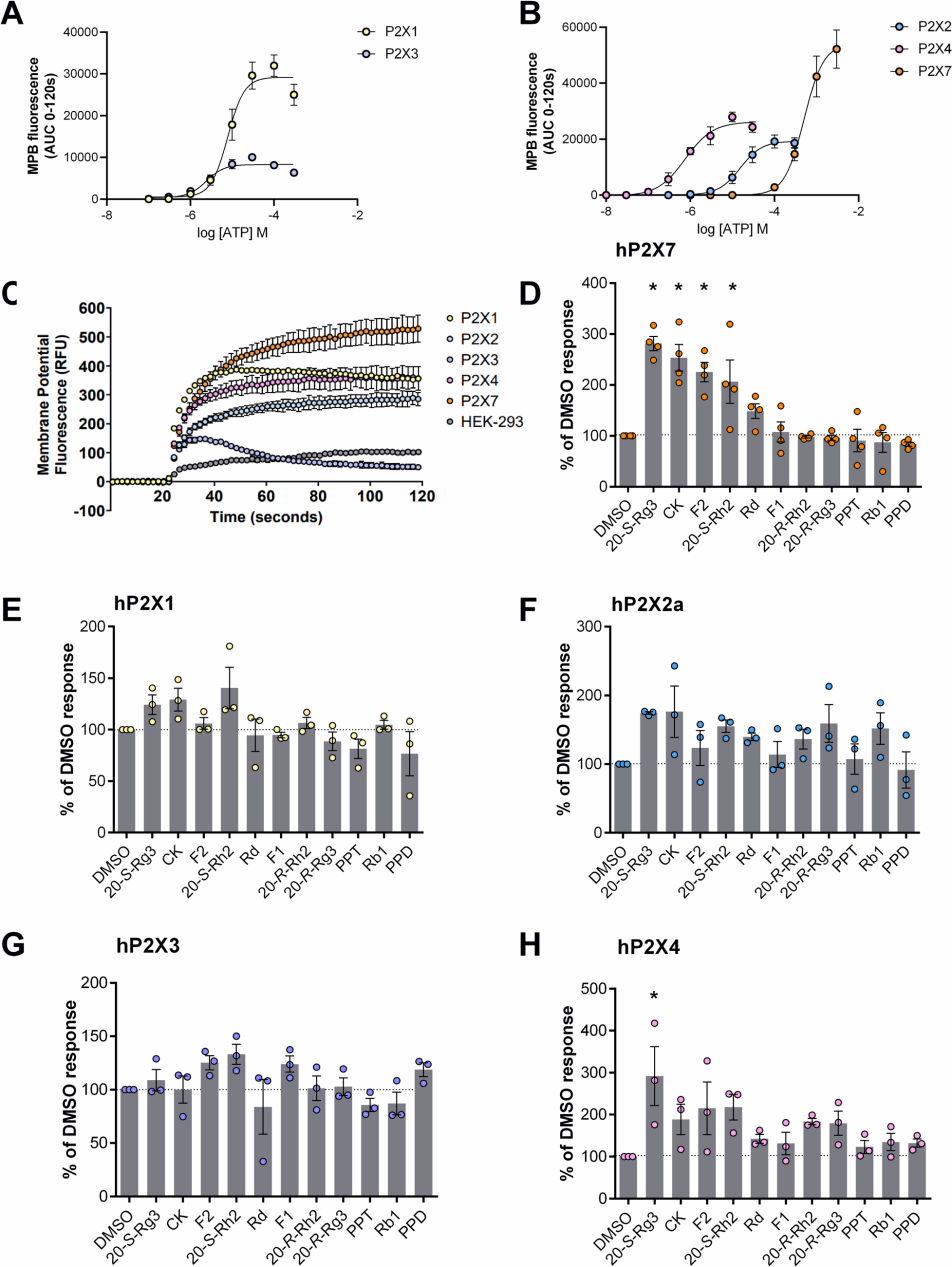
Membrane potential assay responses for human P2X receptors. Concentration response curves for ATP are shown for **A** P2X1 and P2X3, **B** P2X2a, P2X4 and P2X7. Responses were baseline corrected and AUC was calculated using Softmax Pro v5.4 software. Data is from 3 independent experiments performed with triplicate wells. Curves are fit using a four-parameter non-linear regression in GraphPad Prism. (**C-H**) All ginsenosides were screened at 10 µM using a dual injection protocol (modulator was injected 60 s before agonist). ATP was used at approximate EC_50_ concentrations for each receptor; **D** hP2X7 200 µM, **E** hP2X1 10 µM **F** hP2X2a 10 µM **G** hP2X3 10 µM **H** hP2X4 300 nM. Responses were baseline corrected and AUC calculated using Softmax Pro v5.4 software then normalised to the vehicle control. Data is from 3—4 independent experiments performed with triplicate wells. One-way ANOVA was performed with Dunnett’s multiple comparison test, * indicates P < 0.05

Firstly, we optimised the membrane potential blue assay for measuring P2X responses to the primary agonist ATP. A Flexstation 3 plate reader was used to measure ATP-induced responses and concentration–response curves were generated from area under the curve (AUC) data. We generated clonal stable cell lines for each of the human receptors (hP2X1, hP2X2a, hP2X3) in addition to our stable cell lines for hP2X4 and hP2X7 to reduce variability from transient transfections. ATP responses were measured in standard extracellular buffer containing 2 mM calcium and 1 mM magnesium (Fig. 1). We did not study hP2X5 or hP2X6 receptors as these are not known to exist as homomeric receptors in vivo [20]. EC_50_ values for ATP were as follows; hP2X1 8.2 ± 2.4 µM, hP2X2a 17.6 ± 4.0 µM, hP2X3 2.5 ± 0.5 µM, hP2X4 0.8 ± 0.26 µM, and hP2X7 638.5 ± 354 µM (from n = 3 independent experiments). A representative trace showing the typical response of each P2X receptor compared to untransfected HEK-293 cells is shown in Fig. 1C. There is a small depolarisation induced by ATP in untransfected HEK-293 cells, likely due to endogenous expression of P2Y receptors activating ion channels, however P2X responses membrane potential changes are much larger. We tested 9 ginsenoside chemicals on HEK-293 cells expressing hP2X receptors using the membrane potential blue assay and standard extracellular buffer to keep the experimental conditions consistent. These ginsenosides (Rg3, Rb1, Rd, Rh2, F2, F1, Ck, PPD and PPT), mostly protopanaxadiol ginsenosides, were chosen due to their demonstrated effect on P2X7 [10]. In total there are approx. 20 different ginsenoside compounds [21] but only protopanaxadiol ginsenosides have activity on P2X receptors. An approximate EC_50_ concentration of agonist was used for each individual P2X receptor, and a dual injection protocol was implemented allowing compound/vehicle injection 60 s before the agonist. All ginsenosides were tested at a final concentration of 10 µM and data in Fig. 1 is presented as % of control. We first confirmed that robust potentiation by protopanaxadiol ginsenosides was seen in HEK-hP2X7 using 200 µM ATP (as in previous studies) to induce ion channel activation. 20-S-Rg3, CK and F2 performed as the best PAMs at hP2X7 (Fig. 1D) which agrees with our previous work where screening was performed using the YO-PRO-1 dye uptake assay [15].

For hP2X4 we have previously reported that ginsenosides CK, Rd, Rb1, and Rh2 displayed positive allosteric modulator activity using fura-2 calcium measurements [14]. This was confirmed in the membrane potential assay, and here we discovered that 20-S-Rg3 and F2 are also effective at potentiating the P2X4 receptor (Fig. 1H). We tested the stereoisomers of Rg3 and Rh2 as we previously reported a striking difference in PAM activity at hP2X7 with only S-enantiomers being active [15]. R-enantiomers displayed some PAM activity at hP2X4 in these screening experiments, although this did not reach statistical significance (Fig. 1H). For hP2X2a we observed increased ATP responses with CK, 20-S-Rg3, 20-R-Rg3, 20-S-Rh2 and Rb1 (Fig. 1F) however, this was variable and not statistically significant. hP2X1 and hP2X3 did not show significant potentiation by any of the ginsenosides tested (Fig. 1E, 1G).

**Fig. 2:**
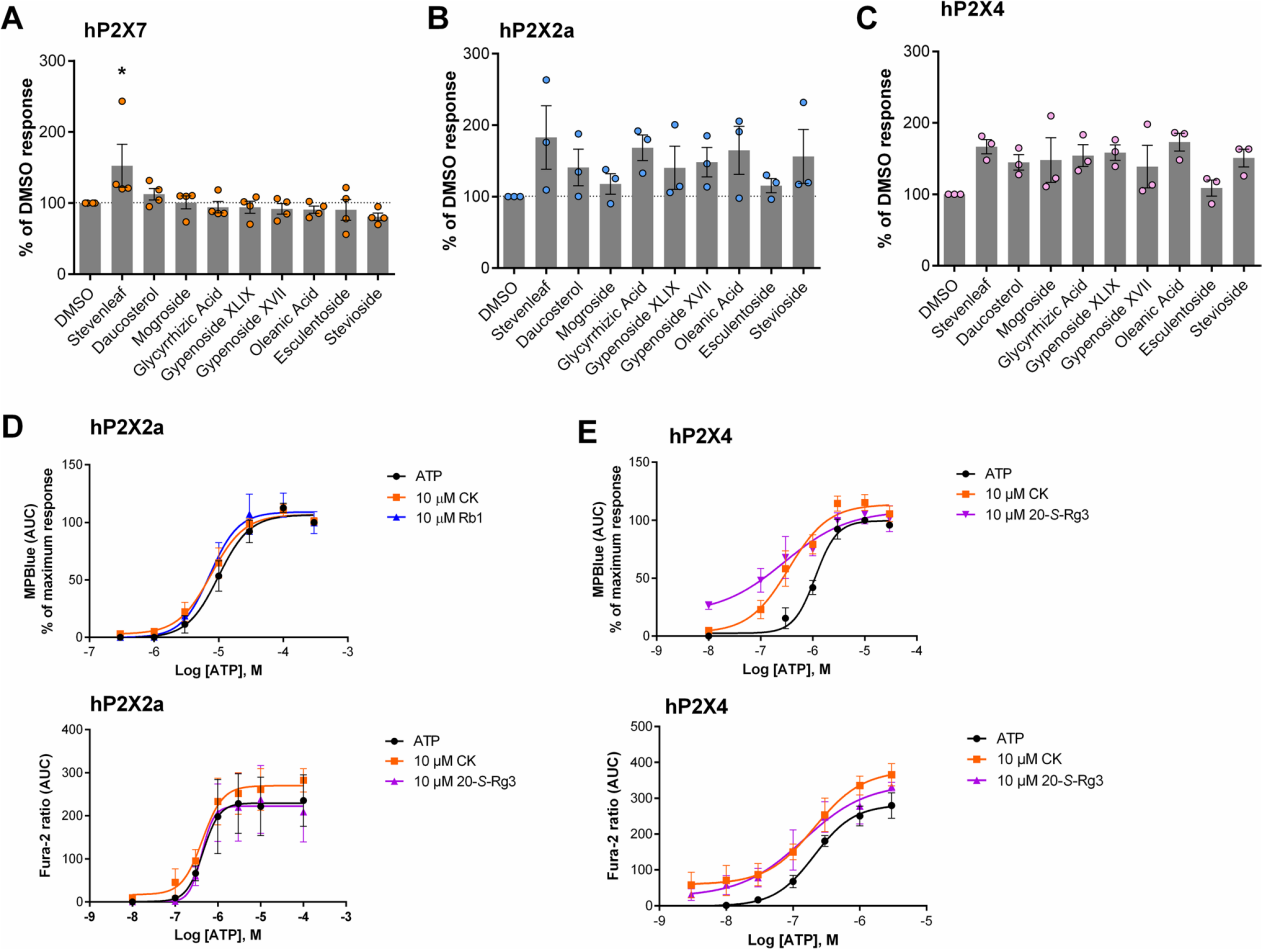
Screening glycosides for positive modulation at human P2X receptors. All glycosides were screened at 10 µM using a dual injection protocol (modulator added 60 s before agonist) using the Membrane Potential Blue assay. ATP was used at approximate EC_50_ concentrations for each receptor; **A** hP2X7 200 µM, **B** hP2X2a 10 µM **C** hP2X4 300 nM. Responses were baseline corrected and AUC calculated using Softmax Pro v5.4 software then normalised to the vehicle control. Data is from 3—4 independent experiments performed with triplicate wells. One-way ANOVA was performed with Dunnett’s multiple comparison test, * indicates P < 0.05. Concentration response curves were generated for ATP at **D** hP2X2a and **E** hP2X4 for both the membrane potential blue assay and fura-2 assay. For Fura-2 experiments cells were loaded with 2 µM fura-2AM and ratiometric measurements (excitation 340/380 nm, emission 510 nm) made using a Flexstation 3 plate reader

Next, we assessed a series of glycosides with similar chemical structures to the ginsenosides. Most of the glycosides did not show robust potentiating activity at hP2X7 in this assay with the exception of stevenleaf (Fig. 2A). Our previous study had demonstrated that gypenoside XLIX, XVII and daucosterol showed PAM activity at hP2X7 although this was seen as an increase in maximum response in the dye uptake assay rather than a shift in the ATP dose response curves [15]. For hP2X2a, there was a lot of variability and none of the glycosides showed a statistically significant increase compared to DMSO control (Fig. 2B). For hP2X4, several glycosides increased the EC_50_ ATP responses including oleanolic acid and stevenleaf (Fig. 2C), although this was not statistically significant.

We further investigated selected ginsenosides at hP2X2a and hP2X4 using concentration response curves to ATP. Using the membrane potential assay, ginsenosides CK and Rb1 induced a minor shift in the ATP concentration response curve at hP2X2a receptors (Fig. 2D). The average EC_50_ for ATP was 12.62 ± 5.4 µM in these experiments and in the presence of ginsenosides CK and Rb1 this was 8.93 ± 4.2 µM and 8.52 ± 3.3 µM respectively. Using a second assay to validate this, fura-2 calcium measurements were made and ATP concentration response curves repeated with the ginsenosides (Fig. 2D). EC_50_ values were similar for all three conditions suggesting no potentiation. Using the membrane potential assay for hP2X4, a left-ward shift in the ATP concentration response curve was observed for ginsenoside CK and S-Rg3 (Fig. 2E) with EC_50_ values shifting from 975 ± 333 nM to 407 ± 302 nM with CK and to 563 ± 572 nM with S-Rg3. Using the fura-2 assay to validate this, ATP concentration response curves were repeated in the presence of the selected ginsenosides. This experimental data validated the potentiating effect of CK and S-Rg3 at hP2X4 (Fig. 2E).

## Discussion

This is the first study where multiple P2X receptor responses have been measured using a single assay for screening purposes. The FLIPR membrane potential assay was easy to use and has potential value in future screening programs. We used the membrane potential blue assay kit from Molecular Devices, however the same kit is available in a red assay utilising a different quenching dye. Others have used the membrane potential assay for investigating P2X1 responses [22] or P2X7 responses [16]. EC_50_ values for ATP at multiple hP2X receptors were similar to those stated in literature [23] and we used this information to select an EC_50_ concentration for screening experiments. With P2X7 we have a wealth of evidence for positive allosteric modulation by ginsenosides [10, 13, 15] and we see similar effects for key protopanaxadiol ginsenosides (CK, F2, S-Rg3, S-Rh2 and Rd) in the membrane potential assay, validating its use for screening. With P2X4, we have previously reported that CK and Rd are effective positive allosteric modulators [14] and here we can report a new finding that S-Rg3 is a good positive modulator at hP2X4 with some potentiation also seen with F2 and S-Rh2 (Fig. 1). For the other P2X receptors, there were no statistically significant results from our screening suggesting that ginsenoside potentiation is restricted to just P2X7 and P2X4 receptors.

There was a minor potentiation of hP2X2 receptors by CK, S-Rg3 and Rb1 (Fig. 1) which we followed up by performing complete concentration–response curves for ATP in the absence and presence of ginsenosides (Fig. 2). CK did induce a small shift in the EC_50_ for ATP which is not hugely dissimilar to the effect of progesterone, an accepted positive modulator for P2X2 [24]. However, this was not validated in the fura-2 experiments. Regardless, the effect on P2X7 and P2X4 is much greater than at P2X2 suggesting the following rank order of effect for ginsenosides: hP2X7 > hP2X4 > > hP2X2 > hP2X1 > hP2X3. Our data confirmed that CK potentiates hP2X4 and revealed S-Rg3 as a highly active ginsenoside. At P2X4 there was also an increase in the maximum response in the presence of CK or S-Rg3 suggesting these positive modulators increase the amplitude of the response in addition to affecting receptor affinity for ATP. Screening of the glycosides at multiple P2X receptors did not yield any new discoveries.

We considered how this data could enhance our understanding of the potential ginsenoside binding site in the central vestibule region, previously postulated by our group in 2019, but not yet confirmed [13]. There is some sequence similarity in the β2 and β14 strands with key amino acid residues identified by us (D318, L320, S60) somewhat conserved in P2X1—4 receptors [13]. If this is the correct location for the ginsenoside binding pocket, there must be other limiting factors preventing potentiation by ginsenosides. For P2X1 and P2X3 receptors, which undergo rapid desensitisation following agonist binding, the lateral portals and central vestibule may not be accessible for long enough to allow significant binding to occur. Further mutagenesis around the predicted pocket and structural studies are needed to understand where positive allosteric modulators bind.

Protopanaxadiol ginsenosides are abundant in *Panax ginseng* extracts and are intensively studied around the world for better understanding traditional medicinal herbal practices [25]. Upon ingesting ginseng preparations, plasma concentrations of ginsenosides are typically in the range 0.1—100 ng/ml [26, 27] which is lower than the concentrations of purified individual ginsenosides tested in our study. However, our pharmacological studies strive to identify novel modulators of P2X receptors that could be further refined through drug development into synthetic agents with relevance for treating disorders where positive allosteric modulation could be beneficial as reviewed in [9].

## Data Availability

All data supporting the findings of this study are available within the paper and raw data is available upon reasonable request to the corresponding author.
